# Gender stereotypes in preschoolers’ mental rotation

**DOI:** 10.3389/fpsyg.2024.1284314

**Published:** 2024-02-05

**Authors:** W. Miro Ebert, Leonardo Jost, Petra Jansen

**Affiliations:** Faculty of Human Sciences, University of Regensburg, Regensburg, Germany

**Keywords:** spatial ability, gender stereotypes, human sex differences, preschool, children, mental rotation, kindergarten

## Abstract

The investigation of gender stereotypes constitutes a relevant approach to understanding the development of spatial ability and sex differences in the domain. This was the first study concerned with the presence of implicit and explicit gender stereotypes about spatial ability, and their potential relation to spatial task performance, in preschool-aged children. Our full sample consisted of 138 4- to 6-year-old kindergarten children. The experimental procedure consisted of three parts. Children completed an implicit association task, a short questionnaire on explicit stereotypes, and a chronometric mental rotation task. Preschool-aged children held explicit gender stereotypes about spatial ability linking it to boys rather than girls. Boys exhibited stronger stereotypes in this regard than girls. We also found evidence for the presence of implicit stereotypes. However, implicit stereotypes were not found in sub-group analyses. No clear relationship between stereotypes and mental rotation performance emerged, but our results suggest that implicit stereotyping affected mental rotation accuracy differently in girls compared with boys. Our main conclusion was that children already hold stereotypic beliefs about spatial ability at preschool age. There did not seem to be a relationship of stereotyping with spatial ability at this age.

## 1 Introduction

Spatial ability entails several skills relevant to various tasks and activities throughout life. For example, young children will employ spatial skills when playing certain sports or games. Around the time they reach school age, spatial demands such as wayfinding emerge. In adulthood, more complex navigation or assembling furniture (Newcombe, 2020) are just a few examples of tasks that rely on spatial ability. Besides its relevance to everyday life activities, it is also suggested that spatial ability plays a significant role in science, technology, mathematics, and engineering (STEM; Newcombe, 2017). Spatial skills predict the tendency to choose an occupation or education in this field (Wai et al., 2009) and the performance or success in pursuing such a career (Newcombe and Shipley, 2014).

Crucially, gender differences in favor of men are observable in STEM enrollment and success (Wang and Degol, 2013) and spatial cognition (Levine et al., 2016). A well-researched aspect of spatial ability is mental rotation (MR), which entails the representation and transformation of an object in mind. The gender differences mentioned above are particularly pronounced in MR tasks (Lauer et al., 2019), and performance on these tasks correlates significantly with relevant STEM outcomes (Khine, 2017). Despite somewhat conflicting findings regarding MR in early childhood, gender differences are consistently found in older populations, for example in children 10–12 years of age or older (Lauer et al., 2019). Both biological and psychosocial mechanisms have been proposed to explain the observed differences. As Hahn et al., (2010a,b) elaborate, there is evidence in support of either explanation and the two are not mutually exclusive. One potential mechanism concerns gender stereotypes. It is a common assumption that stereotypes can causally affect performance. Numerous studies demonstrate stereotype related effects that affect mental rotation performance in adults (e.g., McGlone and Aronson, 2006; Moè and Pazzaglia, 2006). Correlational data also shows that beliefs about gender-specific ability may relate to performance on spatial tasks despite the absence of grave gender differences in task performance (e.g., Sanchis-Segura et al., 2018). Therefore, it is not unlikely that stereotype endorsement may precede notable gender differences in spatial ability and contribute to the later development of said differences.

When discussing gender stereotypes, a distinction between explicit and implicit stereotypes can be made. Explicit stereotypes are typically assessed through self-report measures such as questionnaires. Implicit stereotypes on the other hand, must be measured indirectly without individuals being aware of the constructs or association that a measure targets. The ways in which gender stereotypes may impact spatial ability performance are manifold. Stereotype threat describes a mechanism whereby a group that is subject to a negative stereotype underperforms on tasks that relate to the stereotype (e.g., Schmader et al., 2008). Conversely, stereotype lift is the tendency of a group to perform better on a task when this task is linked to a negative stereotype about an outgroup (Walton and Cohen, 2003). As hinted above, both stereotype threat and lift effects have been demonstrated in various studies with adult participants (e.g., McGlone and Aronson, 2006; Moè and Pazzaglia, 2006). Importantly, Moè and Pazzaglia (2006) found stereotype endorsement to play a role in the emergence of stereotype threat effects. Specifically, women who did not endorse the typical stereotype of male superiority in spatial cognition did not perform worse after receiving stereotype-reinforcing instructions. Moreover, the stereotyped nature of stimuli used in mental rotation tasks affects performance (e.g., Rahe et al., 2020; Rahe and Jansen, 2021). Sanchis-Segura et al. (2018) observed a relationship between implicit gender stereotypes and mental rotation performance. In their study, associating men rather than women with science was negatively correlated with the performance of women but positively correlated with the performance of men. They suggest that stereotypes held by an individual may affect performance through shaping task-related confidence levels.

More indirectly, stereotypes held by parents, educators, and society at large can affect children’s perceptions and beliefs about gender specific behaviors and aptitude and the opportunities they are offered to develop certain skills, such as spatial thinking (King et al., 2021). That is, based on their gender children may be exposed to certain language, toys, or games that do or do not relate to spatial skills. Such exposure would then shape children’s gender stereotypes and of course affects how much they get to practice spatial skills.

As highlighted by King et al. (2021), research on the presence of stereotypes in young children is relatively scarce. Nevertheless, evidence suggests that some forms of stereotyping may be observable in children between the ages of three and four (King et al., 2021). As noted earlier, one approach to measuring stereotypes is through implicit associations, which has been shown to work even in preschool-aged children (e.g., Cvencek et al., 2011a; Qian et al., 2017). A prominent measure in the assessment of implicit associations is the Implicit Association Test (IAT; Greenwald et al., 2022), which is thought to measure the (relative) strength of automatic associations between concepts and attributes/categories. Multiple adaptations of the IAT for use with young children have been developed (e.g., Thomas et al., 2007; Cvencek et al., 2011a,b). At the core of these adaptations lie simplifications of the task, for example through reduction of the number of trials, the use of larger response keys and changes to the modality in which stimuli are presented. Specifically, these tests often rely on pictures as stimuli rather than words. When words are used, the visual presentation is accompanied by an audio recording of the word being read out loud (e.g., Cvencek et al., 2011a,b).

Gender stereotypes relating to food (Graziani et al., 2021), implicit racial bias (Qian et al., 2017), and other associations (e.g., Cvencek et al., 2011a) have been studied in preschool children as young as 4-years-old using adapted IATs. The findings of Graziani et al. (2021) are of particular interest in the context of the present study since they pertain to gender stereotypes. In their research, boys showed implicit gender stereotypes linking meat to boys. Girls did not exhibit implicit stereotypes, and neither boys nor girls demonstrated explicit stereotypes in this regard Graziani et al. (2021). This highlights that specific implicit gender stereotypes may be evident at a young age despite an absence of explicit stereotyping. Potentially, stereotypes begin to form without children being consciously aware of them Graziani et al. (2021) and before they are used to inform behavior. However, explicit and implicit measures may not always tap the same associations, hence such inferences should be considered cautiously. In any case, the findings of Graziani et al. (2021) suggest that measuring gender stereotypes in preschool using an adapted IAT would be feasible.

Findings regarding the emergence of stereotypes about spatial skills are somewhat heterogeneous. Generally speaking, there is evidence of explicit and implicit gender stereotypes concerning spatial ability in children as young as 10 years of age (Neuburger et al., 2015; Heyden et al., 2016; Moè, 2018). However, some findings suggest that implicit (Heyden et al., 2016) and explicit (Neuburger et al., 2013) stereotypes are only held by boys at this age. In the context of the current study it is important to note that arguably, the implicit measure used by Heyden et al. (2016) did not exclusively tap into stereotypes concerning spatial ability. Some of the words used to represent the concept (*numbers, sums*) are likely also related to the field of mathematics. Taking the discussed findings and potential measurement impurity into consideration, it is unclear whether stereotypes regarding spatial ability are held by both girls and boys in this age group.

In sum, a knowledge gap remains regarding spatial ability related gender stereotypes in younger children and the concrete relationship between stereotypes and mental rotation performance. Interesting and crucial questions, such as when these stereotypes begin to form/emerge, remain open.

Another social factor which is related to spatial ability and gender differences in this domain is socio-economic status (SES). Ruthsatz et al. (2012) found positive correlations between parental SES, measured through parents’ educational level, and performance on a paper-pencil mental rotation test in both boys and girls in fourth grade. Specifically, in boys maternal- and paternal SES related to test performance and in girls the combined SES (highest education level between the parents) related to test performance. Interestingly, Levine et al. (2005) found gender differences on two spatial tasks only in children from middle- and high SES families but not in those from low SES backgrounds. In their study, children from higher SES backgrounds overall outperformed those from lower SES backgrounds. The authors propose that boys and girls from low SES backgrounds may enjoy equally little stimulation (e.g., playing with Legos) that would foster spatial ability and boys from higher SES backgrounds engage more with spatially relevant stimuli compared to their female counterparts (Levine et al., 2005). Besides the relationship with spatial ability, there is also evidence to suggest links between SES and gender stereotypes. Lily (1994) found that paternal SES related positively, and maternal SES related negatively to girls’ stereotypes about computer users. Neither paternal- nor maternal SES related to boys’ stereotypes. Similarly, Ruthsatz et al. (2012) observed a negative correlation between maternal SES and girls’ gender stereotypes but no relation between parental SES and boys’ gender stereotypes. It should be noted that these studies were conducted in school children.

This study aims to illuminate parts of the uncertainty regarding explicit and implicit gender stereotypes related to spatial ability in early childhood, as well as their potential relation to MR performance. Implicit associations of spatial toys with gender will be measured using an adapted preschool IAT. Moreover, explicit stereotyping will be assessed using a questionnaire concerned with the suitability of spatial activities, based on a questionnaire used by Heyden et al. (2016). To investigate the potential relationship between stereotypes and MR performance, we will compute correlations to relate implicit and explicit stereotyping to MR performance and examine the main effects of stereotypes and possible interaction terms to understand the potential differences in how stereotypes may affect MR performance between the sexes. We will also collect data on parental SES and examine potential relations of this variable with MR performance and gender stereotypes. SES will also serve as a covariate in our analyses. The related hypotheses are the following.

*H1.1*: Children will hold implicit and explicit gender stereotypes concerning spatial toys (implicit) and spatial play activities (explicit), linking them to boys rather than girls. We expect that children’s early experience with toys will be reflected in the early emergence of related stereotypes.

*H1.2*: Older children will demonstrate stronger associations in this regard. This hypothesis is based on findings showing that five-year-old children displayed more stereotyped play, which constitutes one way of operationalizing stereotype endorsement, than three- and four-year-old children (see King et al., 2021).

*H1.3*: Based on the findings of Heyden et al. (2016), we expect that boys will demonstrate stronger associations in this regard.

*H2*: In accordance with Sanchis-Segura et al.’s (2018) findings, we expect that stereotype endorsement will relate to MR performance such that a stereotype suggesting greater spatial ability of the own gender will be linked with better performance.

We additionally formulated secondary hypotheses concerned with the relationship between parental SES, which will serve as a covariate in our analyses, and MR performance as well as stereotype endorsement. We predict that SES will show a positive correlation with MR performance (S1) and that Components of SES will show a partial relation with gender stereotypes (S2). In line with Ruthsatz et al. (2012) and Lily (1994), we assume that boys’ gender stereotypes will not correlate with parental SES and girls’ gender stereotypes will correlate negatively with maternal SES.

## 2 Method

### 2.1 Participants

Based on an *a-priori* power analysis using G*Power it was estimated that a sample of 109 participants would yield adequate power (1-ß = 0.9) to detect effects of small to medium size (i.e., *f*^2^ = 0.08) at α = 0.05. A total of 138 preschool-aged kindergarten children^1^ (mean age: 5.7 years, SD = 0.47, range: 4.17 to 6.83) whose parents gave their written, informed consent participated in the study. Our targeted age group were five- and six-year-old children. However, three children were 4 years old, two of them were less than a month away from turning five and one child was 50 months (i.e., 4.17 years) old. Sixty participants were girls (mean age: 5.7 years, SD = 0.43) and 78 were boys (mean age: 5.6 years, SD = 0.50). One participant was excluded from analyses that included implicit stereotype score, due to an error rate greater than 35% on the IAT. Against our expectations, approximately 28% of participants (39 individuals) had to be excluded from analyses pertaining to MR performance. Specifically, 33 participants were excluded because their accuracy on the MR task was equal to or lower than 50%. Six children decided to terminate their participation before completing any experimental trials on the MR task. Accordingly, our sample for analyses regarding MR performance comprised 98 kindergarten children (mean age: 5.7 years, SD = 0.49, range: 4.17 to 6.83 years) of which 40 were girls (mean age: 5.8 years, SD = 0.45) and 58 were boys (mean age: 5.7 years, SD = 0.52). According to the same calculations outlined above (using G*Power) this sample size resulted in a power of 1-ß = 0.87. Considering that we used generalized linear mixed models (GLMMs) to analyze mental rotation data, this is likely a conservative estimation of the actual statistical power. For economic reasons and the sake of feasibility we decided to terminate data collection even though our target sample size was not met. All participants were recruited through kindergartens in the city and district of Regensburg, Germany. In return for participation the children received little presents and the kindergartens additionally received financial compensation, 150€-200€ depending on the number of children that had participated. Ethical clearance for the study was granted by the Ethics Board of the University Clinic of Regensburg (protocol number: 22–3056-101) and the study was preregistered with the open science framework: https://osf.io/7gtkn/.

### 2.2 Materials

The study was run on a Lenovo Thinkpad laptop computer with a 15.6-inch monitor (1920 x 1080px) placed approximately 60 cm in front of participating children. Two large, colored buttons were used by the children to respond to stimuli. One of these buttons was placed to either side of the computer. Laminated printouts of MR stimuli were used in explaining the MR task to participants. The opensource software OpenSesame (version 3.3.12; Mathôt et al., 2011) was used to implement the experimental procedure.

#### 2.2.1 Implicit stereotypes measure

**Figure 1:**
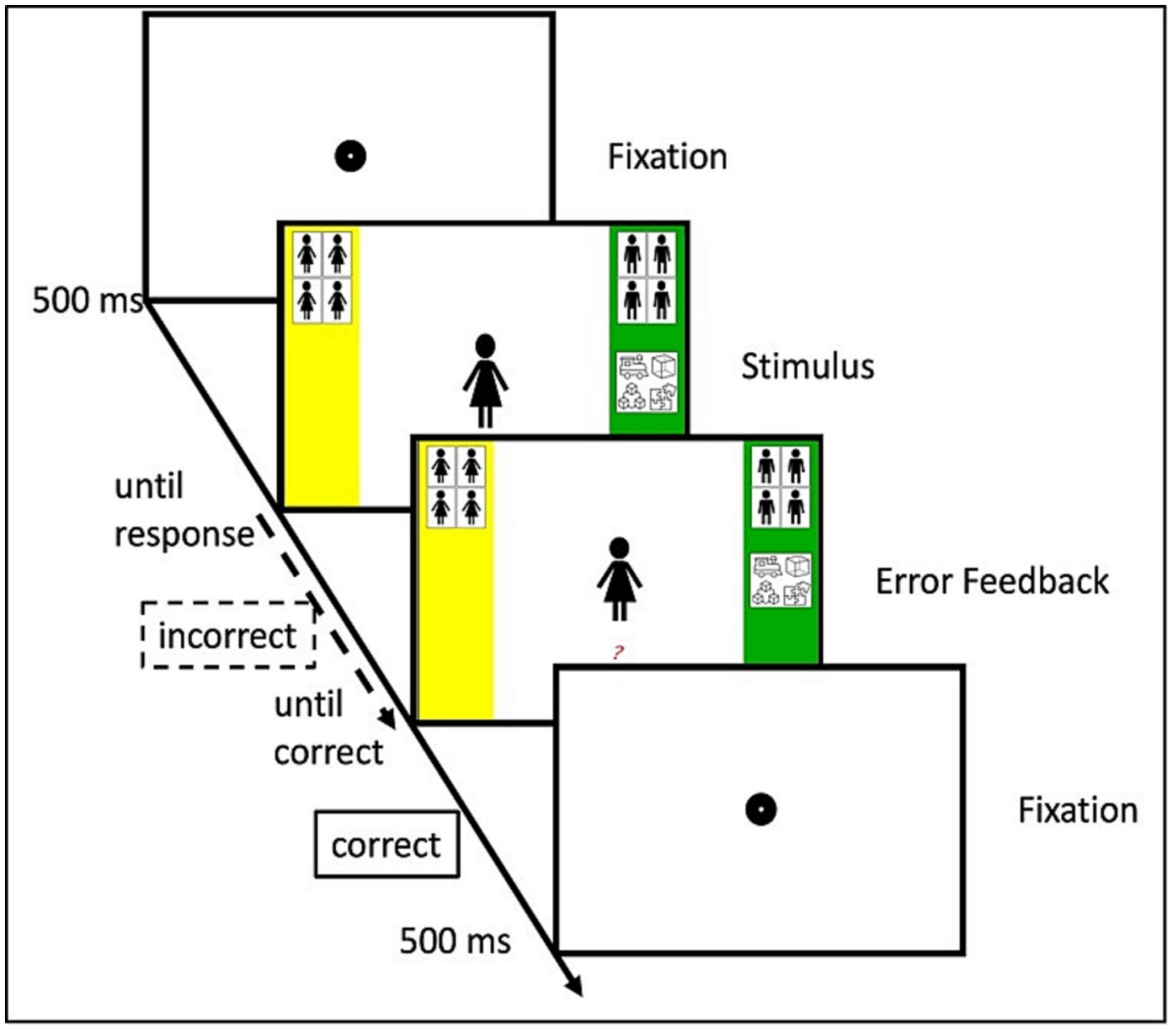
Schematic example of an IAT trial. Error feedback was not displayed if initial answer provided was correct.

An adapted single target IAT (ST-IAT; Wigboldus et al., 2005; *cf.* Karpinski and Steinman, 2006) was administered to measure children’s associations between gender and toys commonly used in spatial play (Lego, building blocks, puzzle, toy train). Block order and response assignment were counterbalanced. Compared to standard IAT procedures, there is only one target category in ST-IATs (in the case of the current experiment, spatial toys). The adaptations of the ST-IAT used in this study (e.g., use of large response buttons, presence of visual reminders at sides of the screen) were primarily based on the work of Cvencek et al. (2011a). Gender was represented by images depicting faces of boys and faces of girls. Toys used in spatial play were also presented in form of images. Over five blocks (three practice blocks, two critical blocks), participants completed 144 trials sorting faces and images of toys. During the first practice block, children completed 16 trials, sorting only faces. In the second practice block, they completed 16 trials sorting both faces and toys. At this, the toys shared a response button with the faces of one of the two genders, for example boys and toys shared the left response button, and girls were assigned the right response button. This practice was followed by a critical block, consisting of 48 trials, in which faces, and toys had to be sorted in the same way as in the previous block. A third practice block of 16 trials followed, in which the response assignment for the toys changed. That is, if boys and toys shared the same response button before, girls and toys shared the same response button in this block. The second critical block consisted of another 48 trials and used the same response assignment. Upon providing a wrong answer a red question mark appeared underneath the stimulus display. Children were instructed that if this happened, they had to correct their answer to continue with the task. This mechanism constituted a built-in error penalty since response times were recorded when the correct answer was given (Cvencek et al., 2011a). After the correct response was given a fixation dot was presented during a 500 millisecond (ms) intertrial interval. A schematic representation of an exemplary trial on this task can be found in Figure 1. ST-IAT scores, called D-scores, were calculated by subtracting the mean latency in the *boys* + *toys* block from the *girls* + *toys* block (see, e.g., Bluemke and Friese, 2008). Accordingly, positive scores indicated a stereotype in favor of boys, and negative scores indicated a stereotype in favor of girls. For analyses pertaining to the relationship between stereotypes and mental rotation, these scores were reversed for girls (see statistical analyses) so that all scores reflected an individual’s stereotype toward their own gender. We did so, since we expected stereotypes favoring the own gender to positively relate to mental rotation performance.

#### 2.2.2 Explicit stereotypes measure

We adapted a questionnaire used by Heyden et al. (2016) to only include items suitable for kindergarten children. The questionnaire was concerned with whom certain spatial activities are more suitable for. For example, “Who does it suit better to play outside?”. Response options were *boys*, *girls,* or *both*. The questionnaires consisted of 21 questions which were presented in random order. The questions and answer options were read aloud to the children by the experimenter and answered verbally by the children. Responses were logged by the experimenter using the laptop computer. An answer indicating the activity was most suitable for boys was scored +1, both was scored 0, and girl was scored −1. The overall score was computed as the mean of response values. Positive scores indicated a stereotype favoring boys and negative scores indicated a stereotype favoring girls. For parsimony, this was called the explicit stereotype score, ESS. For analyses pertaining to the relationship between stereotypes and mental rotation, these scores were reversed for girls (see statistical analyses) so that all scores reflected an individual’s stereotype toward their own gender. We did so, since we expected stereotypes favoring the own gender to positively relate to mental rotation performance.

#### 2.2.3 Mental rotation task

**Figure 2:**
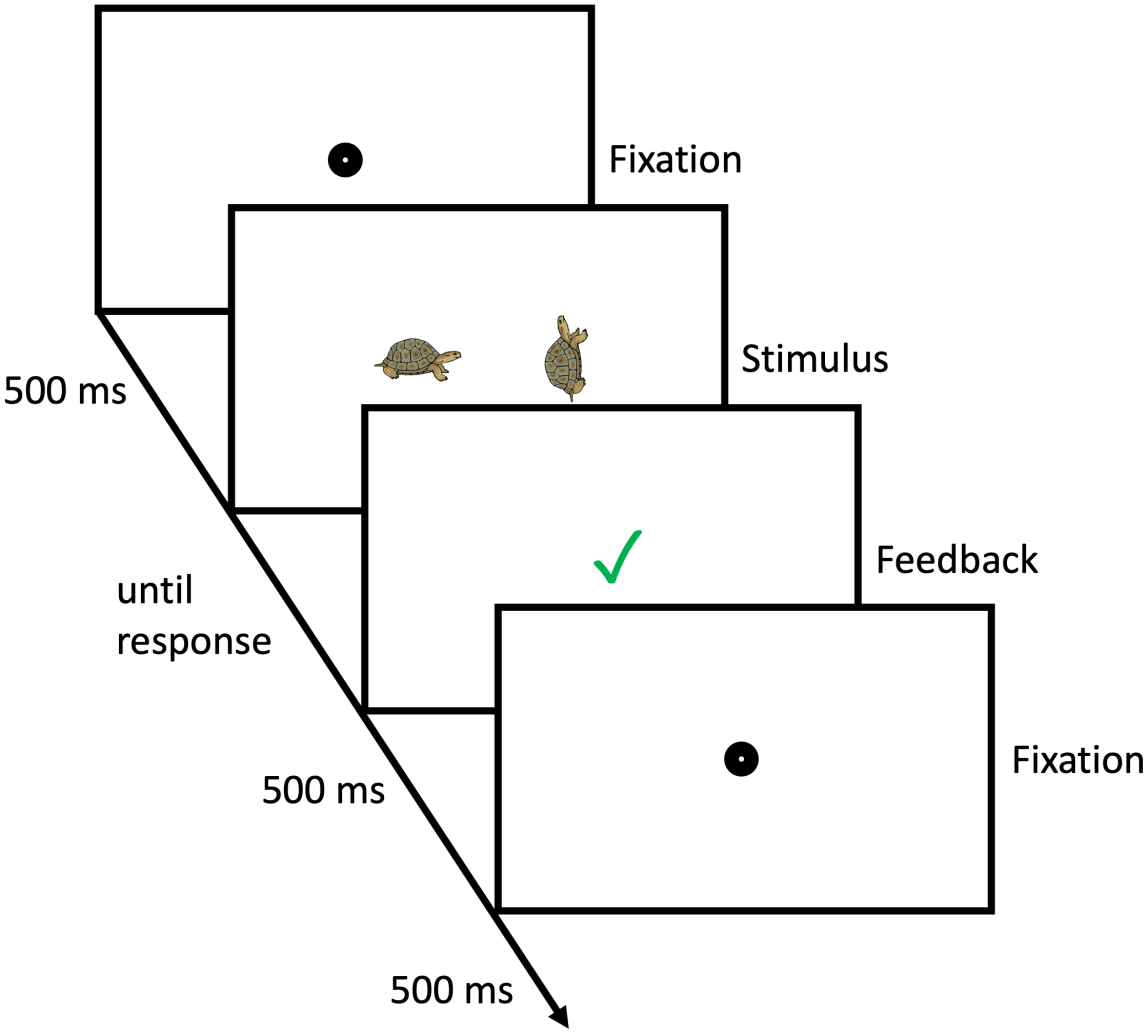
Example of a mental rotation trial.

Participants completed a chronometric animal MR task on a laptop computer. Colored drawings of 12 different animals (camel, crocodile, dog, donkey, elephant, grizzly, lion, pig, rhino, sheep, turtle, and zebra; taken from Rossion and Pourtois, 2004) were used as the experimental stimuli. In each trial two drawings of the same animal were presented simultaneously. The drawing on the left always showed the animal in an upright position facing either to the left (grizzly, elephant, dog, crocodile, rhino, sheep) or the right (donkey, camel, lion, turtle, pig, zebra). The drawing on the right, the comparison stimulus was either the same which means, facing in the same direction when upright to the picture on the left or mirror-reversed, which means facing in the opposite direction when upright. Comparison stimuli were presented upright or rotated by 90°, 180°, or 270°. To indicate that the two drawings were the same, participants pressed a large yellow button placed to the left of the computer keyboard. Conversely, a large green button on the right-hand side was used to indicate that stimuli were mirror images. Each pair of stimuli was presented until participants gave a response. Participants received feedback after each trial. A green checkmark appeared for 500 ms following a correct response. A red “x” appeared for 500 ms following a false responses. After each trial a fixation dot was presented for 500 ms before the next trial began. An exemplary trial is displayed in Figure 2. Sixteen practice trials using pictures of a leopard and deer as stimuli preceded the critical trials. There were 96 critical trials (12 animals * 2 orientations * 4 angles of rotation) in random order such that each combination was presented exactly once. Participants were afforded the opportunity to take a break after every eight trials. After 48 trials all children took a mandatory 2–5-min break. Response time and correctness of responses were logged. A slightly different version of this task has already been used in previous work with kindergarten children (Hahn et al., 2010a). Mental rotation response time and accuracy were used as outcome variables to examine our second hypothesis.

#### 2.2.4 Socio-economic status questionnaire

Parents of participating children were asked to voluntarily provide information regarding their socio-economic status (SES). Providing this information was, however, not a prerequisite for their child’s participation. The questionnaire used was based on the work of Lampert (2018) and pertained to educational background, professional status, and income. In each of these categories a maximum score of 7 could be obtained. The overall index was calculated as the sum of the sub scores. Some parents did not provide information regarding their income. Where this was the case, the value was imputed as the mean income of other individuals whose education and profession scores were the same as those of the person with a missing value. More detail and a scoring table can be found in Lampert (2018).

### 2.3 Procedure

The experiment was conducted by three experimenters, one of whom was female and the other two were male. Participants were tested individually in a quiet room at their respective kindergartens. Consent forms and SES questionnaires filled in by the parents were given to the experimenter by kindergarten staff. Before the experiment began, the general procedure was explained to the children, and they were asked whether they wanted to participate. If they indicated that they would like to participate, they were asked to color a flower serving as a replacement for a classical signature on a consent form. The experimental sequence was the same for all participants. First, they were introduced to the ST-IAT as a game in which they would need to sort images. Each block was preceded by corresponding instructions outlining which stimuli required which response in the respective block. In between blocks, children were encouraged to take breaks. After completing the ST-IAT a short break was implemented. After the break children were told that the experimenter would like to ask them some questions to which there were no right or wrong answers. The experimenter then proceeded to read the questions to the children and record their responses. The questionnaire was followed by a short break after which the MR task was explained to participants. The decision they had to make was illustrated using printouts of the leopard stimulus which could manually be rotated by the children. Beginning with presentation of two manipulable printouts both showing the animal in an upright position either facing in the same or different directions the experimenter explained that the two could either be identical or different (i.e., mirror-reversed). The experimenter then presented several different configurations in which the stimulus on the left would always be upright whereas the stimulus on the right was rotated in most instances. Children were allowed to manually rotate the right printout to determine whether the images were the same or mirror-reversed. Once children appeared to have understood the task, they moved on to the computerized practice trials and the critical trials afterwards. In some rare instances, it became apparent during the computerized practice trials that children had not yet understood the task well. In these cases, additional explanations using the cut-outs were given by the experimenter. For all computerized trials, they were instructed to respond as quickly and accurately as possible. At the end of the experiment, children were thanked for their participation and given a sticker sheet. The duration of the described procedure was approximately 45 min per participant.

### 2.4 Statistical analyses

Outliers in mental rotation response time were excluded on within subjects basis. Specifically, response times two standard deviations below or above the mean response time per angle of an individual were excluded from response time analyses. Additionally, response times faster than 300 milliseconds were excluded from all mental rotation analyses. This procedure was not preregistered but implemented because experimenters frequently observed quick responding when participants appeared to lose focus or pressed a button immediately following a pause screen.

To determine whether children held implicit or explicit gender stereotypes related to spatial ability, linking this quality to boys rather than girls, we conducted one-sample t-tests for each stereotype measure. To test whether older children held stronger stereotypes (Hypothesis 1.1), and whether the degree of stereotyping varied between genders (Hypothesis 1.2), we ran two separate two-way ANCOVAs with the stereotype measures as the dependent variables, age in years, and gender as the independent variables, and SES as a covariate. Our secondary hypotheses (S1 and S2) pertaining to SES were tested through the calculation of correlations between SES and MR outcomes and stereotype scores.

To examine our second hypothesis, we calculated correlations between stereotype scores and MR outcomes. Since we predicted that a positive stereotype toward the own gender would be linked with increased performance, we reversed ST-IAT scores, and ESS for girls. We also fit linear mixed models (LMMs) and GLMMs, where MR response time and MR accuracy served as the dependent variables, to our data using the lme4 package (version 1.1.34; Bates et al., 2015) in R (version 4.2.2; R Core Team, 2022). Per dependent variable, a (G) LMM was fit to two distinct datasets, one dataset including our full sample (*N* = 98) and one including only those participants for whom SES data were available (*N* = 83). ST-IAT score, explicit stereotype score, gender (male, female), age (in months), and rotation angle (0°, 90°, 180°) were included as independent variables. ST-IAT scores and ESS were reversed for girls, in this analysis. To facilitate interpretation of main- and interaction effects, gender was coded at −0.5 (female) and 0.5 (male). Gender was entered as a factor, while all other variables were treated as continuous variables and scaled based on their respective standard deviations to facilitate model convergence. Interaction terms of gender and each of the stereotype measures were included in each model to explore whether the relation between stereotyping and MR varied between boys and girls. In the models fit to the reduced dataset, normalized SES score was additionally included as a covariate. The random factors participant, institution (i.e., the specific kindergarten), and stimulus were included in all models before model reduction to “(1) estimate the extent to which mean responses vary across units of the random factor (s); (2) allow inferences about whether fixed effects generalize beyond the units sampled in the random factor (s); (3) remove variability in responses that are associated with the random factor (s) rather than the conditions of experimental interest (i.e., reduce Type I error rate)” (Lo and Andrews, 2015, p. 5).

Accuracy data were analyzed using GLMMs based on the binomial distribution as recommended by Dixon (2008). Trials in which MR stimuli were identical and responses were not faster than 300 ms were used for these analyses. For analyses of response time data, our preregistered plan was to follow the recommendations of Lo and Andrews (2015) and we compared a gaussian, an inverse gaussian, and a gamma distribution on the most complex models. Although models following the gamma distribution fit the data best, these models were tied to convergence issues even after reduction of the random effects structures. Therefore, we opted for the use of linear mixed models (LMMs) instead. Only correctly solved trials on which stimuli were the same were used in response time analyses. Model building was based on research by Barr et al. (2013), Bates et al. (2015), and Matuschek et al. (2017). Guided by their recommendations, we started from models that included all fixed effects, random intercepts, and random slopes for angle of rotation. When applicable we reduced the random effects structure in a backward selection procedure. Based on likelihood ratio tests (LRTs) we determined whether random correlations and random slopes could be excluded from a given model without a significant decrease in model fit. At this, we began with the random correlations and excluded those effects yielding the least significant decrease in model fit one by one. Based on recommendations by Matuschek et al. (2017) we excluded a random effects component when its removal was associated with a *p*-value greater than.2. However, neither model that was obtained following this approach did converge. Therefore, models were further reduced in a backward procedure removing components one by one, based on which component had the greatest *p*-value associated with its exclusion, until a converging model was found. If two or more equally parsimonious models converged, the better of the converging models was selected as the final model. If at a certain level of complexity only one model converged, that model was the final model. Information regarding the random effects included per model can be found in the results section. To test for significance of the included fixed effects we employed LRTs comparing final models to models excluding the fixed effect in question.

## 3 Results

### 3.1 Stereotype endorsement

Including all available data per measure, children displayed both implicit gender stereotypes *t* (136) = 1.99, *p* = 0.024, *d* = 0.17 and explicit gender stereotypes *t* (137) = 12.80, *p* < 0.001, *d* = 1.09 in favor of boys. No evidence of implicit stereotyping was found conducting the same t-test using only data of participants who completed the full experiment and were not excluded from analyses into MR outcomes *t* (97) = 1.24, *p* = 0.108, *d* = 0.13. The t-test associated with explicit stereotypes remained significant in the smaller sample *t* (97) = 11.40, *p* < 0.001, *d* = 1.15. Analyzed separately per gender, there was no evidence of implicit stereotypes in girls *t* (58) = 1.29, *p* = 0.102, *d* = 0.17 or boys *t* (77) = 1.51, *p* = 0.067, *d* = 0.17. Explicit stereotypes were found in girls *t* (59) = 5.35, *p* < 0.001, *d* = 0.69 and boys *t* (77) = 14.26, *p* < 0.001, *d* = 1.61. There was no evidence to suggest that older children held stronger implicit *F* (1, 110) = 0.57, *p* = 0.451, η_p_^2^ < 0.01 or explicit *F* (1, 111) = 0.06, *p* = 0.811, η_p_^2^ < 0.01 stereotypes compared with younger children. Results revealed that boys held stronger explicit stereotypes linking spatial activities to boys than girls did, *F* (1, 111) = 46.90, *p* < 0.001, η_p_^2^ = 0.29. No gender difference was observed regarding implicit stereotypes, *F* (1,110) = 0.25, *p* = 0.620, η_p_^2^ < 0.01. These results remained unchanged when rerunning the same analyses in the dataset containing only complete data. Neither explicit stereotype score nor implicit stereotype score was significantly correlated with SES, maternal SES, or paternal SES (all *p*-values >0.075). This held true when correlations were computed for the full sample and separately per gender.

### 3.2 Mental rotation

Descriptive statistics of our study variables and correlations among them are displayed in Table 1.

**Table 1:** Descriptive statistics and correlations among study variables.

Variable (N)	Mean	SD	Correlations (df)					
			2	3	4	5	6	7
1. IAT (137)	34.99	206.04	−0.05 (135)	–	–	−0.17(96)	−0.13(96)	−0.05(111)
2. ESS (138)	0.27	0.25	–	–	–	−0.11(96)	−0.01(96)	0.15(111)
3.IAT reversed (137)	7.89	208.86		–	0.09(135)	−0.03(96)	0.00(96)	0.03(111)
4. ESS reversed (138)	0.16	0.34			–	−0.07(96)	−0.06(96)	0.08(111)
5. MR Accuracy (98)	0.81	0.13				–	0.18(96)	0.21 (81)
6. MR response time (98)	2320.05	779.00					–	0.19 (81)
7. SES (113)	17.46	3.08						–

Correlations between reversed- and non-reversed stereotype scores are omitted.

More detailed descriptive statistics concerning mental rotation response time and mental rotation accuracy are summarized in Table 2. Information on the overall statistics and per gender, and angle of rotation is provided.

**Table 2:** Mean (SD) of mental rotation response time (in ms) and accuracy (proportion of correctly solved items) per gender and rotation angle.

Variable	Sample	Rotation angle			
		Overall	0°	90°	180°
Accuracy	Overall (*N* = 98)	0.81 (0.13)	0.89 (0.15)	0.85 (0.15)	0.64 (0.26)
	Girls (*N* = 40)	0.80 (0.13)	0.89 (0.15)	0.84 (0.14)	0.64 (0.28)
	Boys (*N* = 58)	0.81 (0.14)	0.89 (0.15)	0.86 (0.16)	0.64 (0.26)
Response time	Overall (*N* = 98)	2,320 (779)	1876 (600)	2,299 (835)	2,995 (1269)
	Girls (*N* = 40)	2,373 (749)	1932 (623)	2,319 (788)	3,099 (1187)
	Boys (*N* = 58)	2,284 (803)	1837 (586)	2,286 (873)	2,923 (1329)

Neither implicit nor explicit stereotype score correlated significantly with MR response time or MR accuracy (all *p*-values >0.45). Please note that all stereotype scores reported in this section were reversed for girls (see method). The correlation between SES and mental rotation accuracy was not significant (*r* = 0.212, *p* = 0.054). There was no evidence of a significant correlation between SES and MR response time (*r* = 0.188, *p* = 0.088).

Table 3 contains the model coefficients and associated test statistics of the response time LMM including SES. A significant effect of rotation angle was observed [*χ^2^*(1) = 24.25, *p* = <0.001], with greater angular disparity being associated with longer response times. No other effect reached significance in this model.

**Table 3:** Linear mixed model for the dependent variable MR response time (including SES as covariate).

Predictor	Estimate	SE	95% CI		Test statistic	Value of *p*
Intercept	2832.49	678.38	1417.02,	4230.69		
SES	−15.94	55.62	−131.69,	101.92	*χ*^2^(1) = 0.07	0.787
ESS	84.43	100.91	−119.39,	289.27	*χ*^2^(1) = 0.67	0.412
IAT	−7.64	67.14	−145.30,	133.26	*χ*^2^(1) = 0.01	0.913
Gender	−204.78	179.16	−564.14,	153.82	*χ*^2^(1) = 1.28	0.258
Age	−1158.09	680.44	−2567.62,	266.25	*χ*^2^(1) = 2.56	0.110
Angle	679.63	97.97	476.45,	899.79	*χ*^2^(1) = 24.25	<0.001
ESS * Gender	195.79	206.67	−218.82,	612.77	*χ*^2^(1) = 0.87	0.350
IAT * Gender	41.85	132.09	−226.29,	308.00	*χ*^2^(1) = 0.10	0.756

This model included random intercepts by stimulus, institution, and participant and random slopes for angle by institution and participant.

Model coefficients and associated test statistics of the LMM on response time, excluding SES, are shown in Table 4. Rotation angle was a significant predictor of response time in this model [*χ^2^*(1) = 77.20, *p* < 0.001]. Greater angular disparity was associated with longer response times. There was a significant effect of age in this model [*χ^2^*(1) = 5.00, *p* = 0.025]. Higher age was associated with lower response times. Hence, in contrast to the model of response time including SES, a significant main effect of age was found in this model.

**Table 4:** Linear mixed model for the dependent variable MR response time.

Predictor	Estimate	SE	95% CI		Test statistic	Value of *p*
Intercept	3226.35	646.22	1959.78,	4492.92		
ESS	14.22	95.51	−176.62,	206.14	*χ*^2^(1) = 0.02	0.883
IAT	35.68	61.34	−87.34,	160.25	*χ*^2^(1) = 0.33	0.568
Gender	−173.54	164.82	−504.27,	155.26	*χ*^2^(1) = 1.09	0.297
Age	−1525.78	643.93	−2863.42,	−191.62	*χ*^2^(1) = 5.00	0.025
Angle	648.45	58.71	531.80,	764.76	*χ*^2^(1) = 77.20	<0.001
ESS * Gender	274.44	189.37	−105.53,	659.70	*χ*^2^(1) = 2.02	0.155
IAT * Gender	−104.05	118.58	−345.39,	133.54	*χ*^2^(1) = 0.74	0.389

This model included random intercepts by stimulus, institution, and participant and random slopes for angle by participant.

**Figure 3:**
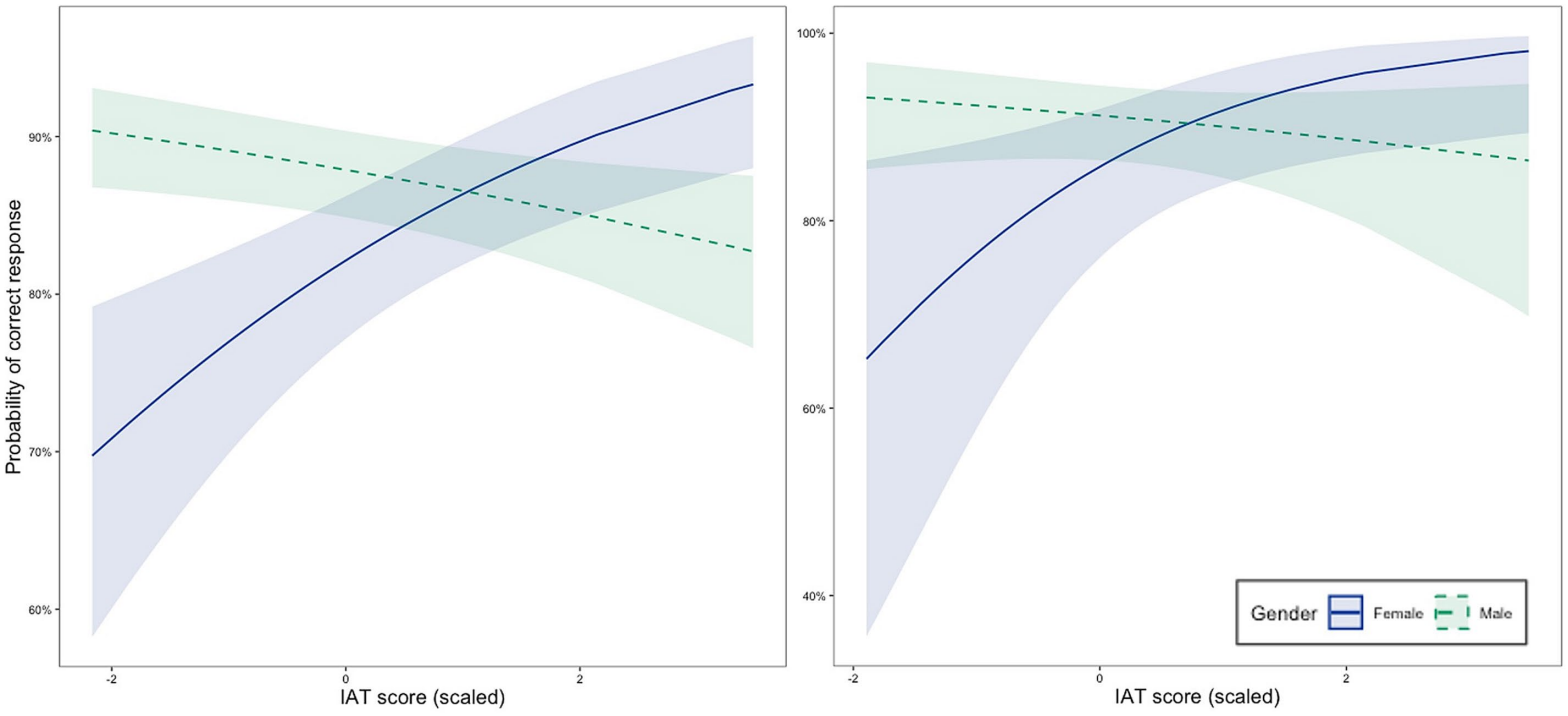
Slopes of IAT per gender. Probability of correct response in mental rotation as a function of gender and scaled IAT score for the full sample (left) and individuals for whom SES data were available (right).

Table 5 contains coefficients and test statistics of the GLMM for accuracy including SES. A significant negative main effect of explicit stereotypes was observed [*χ^2^*(1) = 4.97, *p* = 0.026].^2^ Rotation angle significantly predicted the proportion of correct responses [*χ^2^*(1) = 226.09, *p* < 0.001]. Greater rotation was associated with smaller proportions of correct responses. An interaction of gender and implicit stereotypes [*χ^2^*(1) = 6.13, *p* = 0.013] was evident. Post-hoc analysis revealed that in girls, implicit stereotypes shared a positive relation with mental rotation accuracy [*χ^2^*(1) = 7.00, *p* = 0.008]. In boys there was no significant effect of implicit stereotypes on mental rotation accuracy [*χ^2^*(1) = 0.57, *p* = 0.448]. We plotted the slopes per gender in Figure 3 to facilitate understanding this interaction.

**Table 5:** Generalized linear mixed model for the dependent variable MR accuracy including SES.

Predictor	Estimate	SE	95% CI		Test Statistic	Value of p
Intercept	3.89	1.45	1.00,	6.95		
SES	0.20	0.12	−0.04,	0.45	*χ*^2^(1) = 2.70	0.101
ESS	−0.51	0.23	−0.97,	−0.06	*χ*^2^(1) = 4.97	0.026
IAT	0.24	0.15	−0.06,	0.54	*χ*^2^(1) = 2.46	0.117
Gender	0.79	0.41	−0.03,	1.62	*χ*^2^(1) = 3.61	0.057
Age	−0.59	1.46	−3.52,	2.35	*χ*^2^(1) = 0.16	0.688
Angle	−1.23	0.09	−1.40,	−1.06	*χ*^2^(1) = 226.09	<0.001
ESS * Gender	−0.54	0.46	−1.47,	0.39	*χ*^2^(1) = 1.31	0.252
IAT * Gender	−0.76	0.30	−1.37	−0.16	*χ*^2^(1) = 6.13	0.013

This model included random intercepts by stimulus and participant.

The model for accuracy excluding SES is summarized in Table 6.^3^ As in the model including SES, explicit stereotypes significantly predicted accuracy [*χ^2^*(1) = 9.65, *p* = 0.002]. Angle was a significant predictor of accuracy in this model [*χ^2^*(1) = 251.90, *p* < 0.001]. Again, a significant interaction between gender and implicit stereotypes was found [*χ^2^*(1) = 19.38, *p* < 0.001]. As in the model including SES, post-hoc analyses revealed a significant positive effect of implicit stereotypes favoring the own gender in girls [*χ^2^*(1) = 20.96, *p* < 0.001]. No effect of implicit stereotypes was observed in boys [*χ^2^*(1) = 3.16, *p* = 0.076]. A graph of the slopes per gender can be found in Figure 3. In terms of significance, the only difference compared to the results found when SES was included in the analysis, was a significant main effect of gender in this model [*χ^2^*(1) = 17.06, *p* < 0.001].

**Table 6:** Generalized linear mixed model for the dependent variable MR accuracy.

Predictor	Estimate	SE	95% CI		Test Statistic	Value of p
Intercept	3.53	0.58	2.40,	4.69		
ESS	−0.25	0.08	−0.42,	−0.09	*χ*^2^(1) = 9.65	0.002
IAT	0.10	0.05	−0.00005,	0.20	*χ*^2^(1) = 3.84	0.050
Gender	0.60	0.15	0.31,	0.89	*χ*^2^(1) = 17.06	<0.001
Age	−0.75	0.57	−1.87,	0.36	*χ*^2^(1) = 1.74	0.188
Angle	−1.12	0.07	−1.27,	−0.98	*χ*^2^(1) = 251.90	<0.001
ESS * Gender	−0.31	0.16	−0.62,	0.003	*χ*^2^(1) = 3.76	0.053
IAT * Gender	−0.44	0.10	−0.63,	−0.25	*χ*^2^(1) = 19.938	<0.001

This model included random intercepts by stimulus and institution.

## 4 Discussion

It was the main goal of this study to gather evidence regarding the presence of implicit and explicit stereotypes about spatial ability in preschool-aged kindergarten children and potential relationships of such stereotypes with performance on spatial tasks. To investigate these topics, we conducted a study in preschool-aged kindergarten children and measured both implicit and explicit stereotypes and performance on a chronometric mental rotation task.

### 4.1 Gender stereotypes in preschoolers

Our findings suggest that preschool-aged children already hold explicit stereotypic beliefs about the suitability of activities that involve spatial thinking. Both girls and boys displayed explicit stereotypes in favor of boys. Moreover, we found evidence for the presence of implicit stereotypes favoring boys, in our overall sample. However, the effect size was small (*d* = 0.17) and no evidence of implicit stereotypes, in terms of significance, was found when data were analyzed separately per gender (both *d* = 0.17). The lack of evidence for stereotyping in each gender individually may be due to issues with statistical power, as the effect sizes remain roughly the same. Graziani et al. (2021) found a similar effect size (*d* = 0.19) in investigating implicit gender stereotypes regarding food in preschool children. Indirect support for the hypothesis that preschool-aged children hold implicit gender stereotypes comes from our finding, that implicit stereotypes favoring the own gender related to MR accuracy differently between genders. Hence, we accept our first hypothesis albeit with a note of caution since we could not find evidence of implicit stereotypes in sub-group analyses. There was no evidence in favor of the hypothesis that older children hold stronger stereotypes, be it implicit or explicit, which we therefore do not accept. This is in parallel with findings of Heyden et al. (2016) who did not observe any effects of grade in explicit or implicit stereotypes regarding spatial ability in 10 to 12-year-old children. On the other hand, this finding contrasts the observation that stereotyped play increases with age in preschool children (see King et al., 2021). It remains unclear whether the absence of such an age effect means that explicit stereotypes about spatial ability are fully pronounced at a young age or whether the age range in our sample was too narrow to detect a trajectory. Results regarding gender differences in the degree of stereotyping were mixed. Our findings suggest that boys hold stronger explicit stereotypes than girls, but no difference in implicit stereotypes was observed. Hence this hypothesis that boys hold stronger stereotypes than girls can only be accepted in part. In older children, significant gender differences were observed in both explicit and implicit stereotypes (Heyden et al., 2016). Contrary to our secondary hypothesis S2, we did not observe any correlation between SES and stereotype endorsement. Neither girls’ nor boys’ stereotypes related to maternal-, paternal, or overall SES. This contrasts findings from both Ruthsatz et al. (2012) and Lily (1994), who observed relationships between SES and girls’ stereotypes. However, both studies relied on other methods of assessing SES compared to the present study and were conducted in older children. Whether such relationships are absent in young children, or our methods were not suited to capture them remains unclear.

### 4.2 The relation of gender stereotypes and mental rotation performance in preschoolers

First, our results show a main effect of gender in MR accuracy, only when SES was not accounted for. Other gender effects were not observed. This might suggest that the gender differences in MR accuracy in our sample were better explained by SES. However, since SES data were not available our full sample, it may also be related to statistical power. At large, the absence of gender effects is in line with many other studies in this age group which failed to show a gender difference in this age group on the behavioral level, even though differences have been observed measuring neuronal activity (Hahn et al., 2010a,b). Regarding our hypothesis that children’s stereotypic beliefs would relate to mental rotation performance, the picture is more complex. There is no clear evidence to suggest that implicit or explicit stereotypes relate to mental rotation performance. Contrary to what we expected, explicit stereotypes favoring the own gender were linked to lower accuracy. That is, children who believed that spatial activities fit their own gender rather than the other gender, performed worse than children who believed the opposite. This clearly contradicts our hypothesis H2 and is difficult to embed in the theoretical background. A possible explanation could be that stereotypes are not applied to the self by children in this age group. Apart from this surprising relationship, no other immediate links between stereotypes and mental rotation performance were found. Interestingly, the relationships between implicit stereotypes and mental rotation accuracy varied between the genders. Specifically, more implicit stereotyping in favor of the own gender was associated with higher accuracy in girls and shared no relationship with mental rotation accuracy in boys. On a descriptive level, boys’ accuracy tended to decline with increased stereotype endorsement. Considering these findings, our second hypothesis is rejected. One might assume that boys holding positive stereotypic beliefs about the own gender are well convinced about their own abilities and for this reason give responses in an overconfident manner without much reflection. Girls who believe that girls are more spatially skilled, on the other hand, may place greater emphasis on responding correctly, to demonstrate their skill. However, such explanations remain highly speculative. Another reason might be that girls and boys at this age are unfamiliar with the commonly observed gender differences in mental rotation performance, in favor of boys. So even if they have gender stereotypes regarding the spatial domain, they may not associate these with the mental rotation task which could explain the absence of a clear relationship pattern between stereotypes and mental rotation. This is, for example, different in the context of mathematics in primary school. Primary-school children have experience with the beliefs of teachers and parents regarding the mathematical abilities of boys and girls and the relation between to the own stereotype and the performance in this context is more reliable (Muzzatti and Agnoli, 2007). But there are also studies which did not show a relation between gender-stereotyped beliefs and mental rotation ability in primary school-aged children (Moè, 2018).

### 4.3 Psychosocial explanation and mental rotation performance in preschoolers

Against our expectations we found no evidence of a relationship between SES and MR outcomes. Our secondary hypothesis S1 is, therefore, also not accepted. As outlined earlier, Ruthsatz et al. (2012) did find a relationship between SES and mental rotation performance. There are a few potential reasons for the discrepancy in results. For one, the study by Ruthsatz et al. (2012) was conducted in fourth graders and used a paper-pencil version of a mental rotation task. Moreover, we used a different method of operationalizing SES compared to Ruthsatz et al. (2012). Hence, it is possible that this relationship only emerges when using specific mental rotation tests and operationalizations of SES or it simply develops at a later age. When not accounting for SES, our results indicate a progression in MR performance, regarding response times, with increasing age. A progression of mental rotation ability with age would be in line with a study of Pedrett et al. (2022) who showed that mental rotation ability of asymmetrical shapes continued to develop between 3.5 and 5.5 years. However, when SES is considered, response times do not share a relationship with age. Considering the directionality of age effects in predicting MR accuracy, albeit in the absence of significance, the possibility of a speed-accuracy trade-off should be noted. That is, older children tend to respond faster but descriptively less accurately. Hence, our results do not warrant the conclusion that there was an increase in performance with age, in our sample. Together with the unclear picture regarding the relation between stereotypes and mental rotation in preschool-aged kindergarten children and the absence of gender differences our findings cannot provide support for a psychosocial explanation regarding the emergence of gender differences in mental rotation. The possibility remains, that stereotypes form early and only later exert an influence on spatial skills. Accordingly, to evaluate the relevance of psychosocial explanations of mental rotation performance in children more longitudinal studies with an adequate number of boys and girls in each age group and relevant measurements of social factors like SES or stereotypes and the appropriate mental rotation test for each age group must be conducted (Frick and Pichelmann, 2023).

### 4.4 Limitations

While this study shows the feasibility investigating implicit and explicit stereotypes in the spatial domain in preschoolers and the absence of a clear relationship of stereotypes with mental rotation performance, it has several limitations. First, it is critical to note that the use of questionnaires in children as young as our participants is always difficult and can be problem ridden. We decided to include a questionnaire despite knowing of these hurdles. It was sometimes noticeable to experimenters, that children did not fully comprehend a question, in which case additional explanation was provided. We are aware that such lack of understanding would not always be recognizable, which is part of the reason we decided to discuss the issue. Secondly, we had to exclude a greater proportion of children from our mental rotation analyses, than comparable studies. Potentially, seemingly small differences in task design exerted greater effects than we anticipated. For example, a shorter intertrial interval (500 ms) was used in this study compared to other studies using the same kind of mental rotation task (e.g., Hahn et al., 2010a,b used 2–3 s intervals). Lastly, we have concerns regarding the statistical power to detect certain effects of interest and given the structure of our data were unable to determine whether gender differences in mental rotation accuracy were better explained through SES.

### 4.5 Conclusion

Even though there is clear evidence of sex difference in mental rotation performance in adolescents, the reasons for the emergence of those gender differences are not well understood. This study contributes the novel finding, that explicit gender stereotypes about the spatial domain are held at preschool age. The presence of implicit stereotypes is suggested but doubtful. However, there was no clear relationship between stereotypes held and MR performance. The call for a biosocial approach to investigate mechanisms involved in the development of gender differences in mental rotation is important but its’ realization would likely require large-scale research, for example in the form of multi-center studies.

## Data availability statement

The raw data supporting the conclusions of this article will be made available by the authors, without undue reservation.

## Ethics statement

The studies involving humans were approved by Ethics Board of the University Clinic of Regensburg. The studies were conducted in accordance with the local legislation and institutional requirements. Written informed consent for participation in this study was provided by the participants’ legal guardians/next of kin.

## Author contributions

WE: Conceptualization, Data curation, Formal analysis, Investigation, Writing – original draft. LJ: Conceptualization, Methodology, Validation, Writing – review & editing. PJ: Conceptualization, Project administration, Resources, Supervision, Writing – review & editing.

## Footnotes

^1^Children in our sample were not in compulsory preschool education, although some form of formal education was provided to most of them to facilitate the transition to primary school.

^2^Since we used scores that were reversed for girls in this analysis, this effect suggests that a positive stereotype towards the own gender was associated with lower accuracy.

^3^Due to convergence issues no random intercept per participant was included in this model which may have affected the results.
